# Narrative Review: Gut Microbiota and Its Impact on α‐syn Function in Parkinson's Disease

**DOI:** 10.1002/mbo3.70173

**Published:** 2025-12-05

**Authors:** I. Daniel Salinas‐Velarde, Juan Manuel Donaciano‐Domínguez, Rigoberto Oros‐Pantoja, José Félix Aguirre‐Garrido, Rina María González‐Cervantes, Jacobo Esteban Munguía‐Cervantes, Modesto Gómez López, Jaime Bustos‐Martínez, Alexandra Estela Soto‐Piña

**Affiliations:** ^1^ Facultad de Medicina Universidad Autónoma del Estado de México, Estado de México México; ^2^ Departamento de Ciencias Ambientales Universidad Autónoma Metropolitana‐Lerma, Estado de México México; ^3^ Centro de Nanociencias y Micro y Nanotecnologías, Instituto Politécnico Nacional CDMX México; ^4^ Escuela Superior de Medicina, Instituto Politécnico Nacional, Sección de Estudios de Posgrado e Investigación CDMX México; ^5^ Departamento de Atención a la Salud Universidad Autónoma Metropolitana‐Xochimilco CDMX México

**Keywords:** dysbiosis, gut microbiota, inflammatory processes, nutrition, Parkinson's Disease

## Abstract

Gut microbiota (GM) plays a pivotal role in human health and disease, and its alterations have been implicated in various neurological disorders, including Parkinson's disease (PD). Growing evidence reveals correlations between the abundance of specific bacterial taxa and the severity of motor symptoms and intestinal dysfunction in PD. Moreover, bacterial metabolites have been shown to influence α‐synuclein (α‐syn) aggregation and neurodegeneration. This narrative review aims to explore the current understanding of the gut‐brain axis in PD, specifically the connection between GM and α‐syn function in PD experimental models and patients. Several therapeutic strategies aimed at modulating gut microbiota, such as dietary interventions, fecal microbiota transplantation, and targeted bacterial therapies with the goal of alleviating or preventing PD symptoms, are examined. Understanding the mechanisms through which GM influence neurodegeneration, including inflammation, immune modulation, and microbial metabolite production, offers promising avenues for the development of novel therapeutic strategies targeting the microbiome.

## Graphical abstract

The gut–brain axis links environmental factors, gut microbiota, and α‐synuclein pathology. Altered microbiota promote intestinal α‐synuclein aggregation, which spreads via the vagus nerve to the brain, triggering motor dysfunction. Fecal microbiota transplantation and dietary fiber can alleviate gastrointestinal and motor symptoms in patients and animal models of Parkinson's disease.

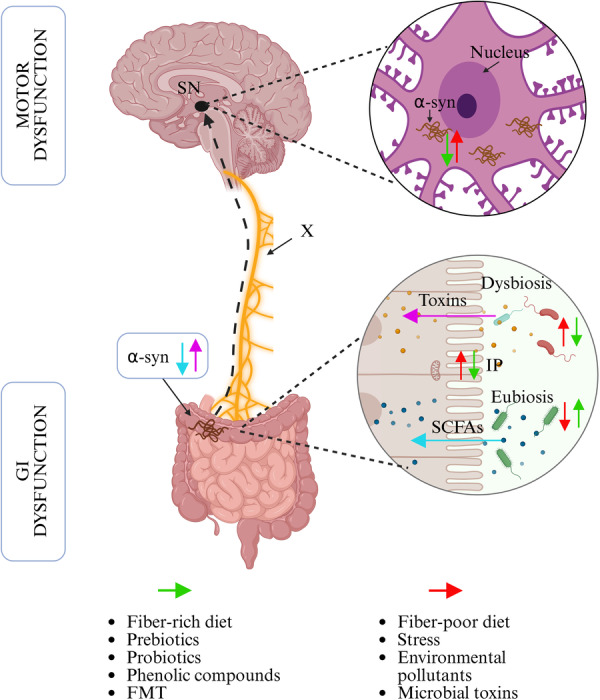

## 1 Introduction

Parkinson's disease (PD) is a progressive neurodegenerative disorder characterized by loss of dopaminergic neurons in the Substantia nigra pars compacta (SN*pc*) and the pathological accumulation of a misfolded protein called α‐synuclein (α‐syn) within these neurons (Kim et al. 2023). According to the Movement Disorder Society clinical diagnostic criteria, the motor hallmarks of PD include bradykinesia, rigidity, and rest tremor (Cosma‐Grigorov et al. 2020). Currently, there is no definitive treatment for PD; however, its symptoms can be managed based on disease severity through pharmacological treatments such as levodopa, monoamine oxidase and catechol‐O‐methyltransferase inhibitors, dopamine agonizts, and amantadine (Cosma‐Grigorov et al. 2020). In addition, emerging preclinical research is exploring the potential of alternative therapies, including the administration of stem cells derived from human umbilical cord blood (Lee et al. 2022).

Although PD is widely considered a multifactorial disease, its exact etiology remains incompletely understood. Increasing evidence implicates a combination of genetic predispositions, environmental factors, lifestyle choices, and disruptions in the gut‐brain axis disruption as risk factors contributing to disease onset and progression (Fang et al. 2024a). The gut‐brain axis is a complex, bidirectional communication network between the gastrointestinal tract and the central nervous system (CNS). This interaction is mediated through neural, endocrine, and immune pathways (Jackson et al. 2022). The gastrointestinal tract harbors a highly diverse community of microorganisms collectively called the gut microbiota, which plays a crucial role in maintaining host health. In recent years, gut microbiota (GM) has been increasingly recognized for its role in modulating the gut‐brain axis and influencing the pathophysiology of multiple chronic degenerative conditions, including PD (Cosma‐Grigorov et al. 2020).

Notably, early gastrointestinal disturbances, such as those caused by exposure to neurotoxic substances or dietary imbalances, have been linked to the emergence of non‐motor symptoms (NMS) in PD, including gastrointestinal dysfunction and systemic inflammation (Fang et al. 2024a). These early alterations in the gut environment can also trigger gut dysbiosis (GD), defined as a disruption in the composition and function of the GM, which is strongly associated with both the onset and progression of PD (Kim et al. 2023). Recent studies have shown that GD promotes the accumulation of α‐syn in the enteric nervous system, which may contribute to the prodromal NMS of PD (Lee et al. 2023). Subsequently, this intestinal α‐syn accumulation is believed to propagate to the brain via the vagus nerve, supporting the hypothesis that gut‐originating pathology can influence central neurodegeneration (Cosma‐Grigorov et al. 2020; Jackson et al. 2022; Shan et al. 2022).

**Figure 1:**
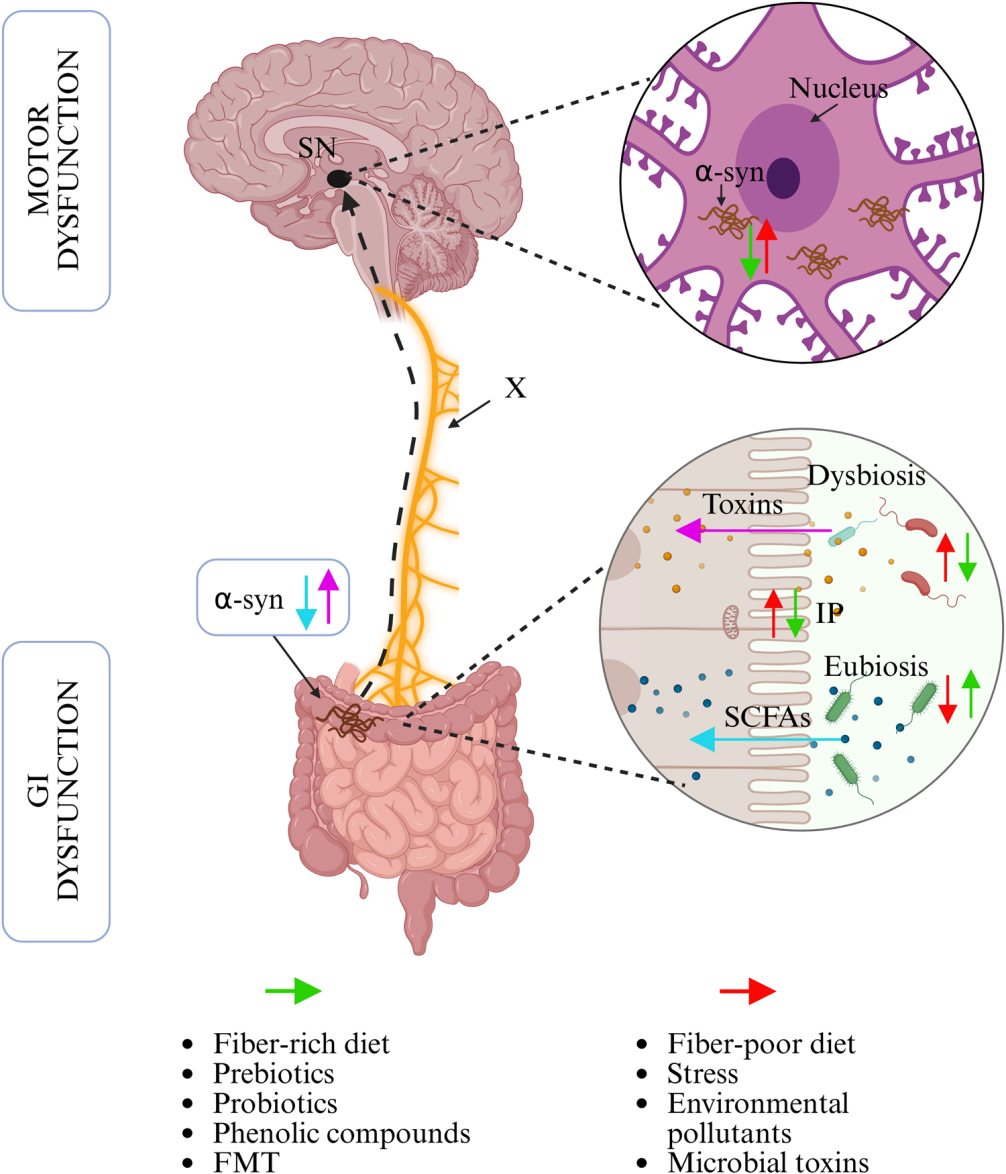
The gut‐brain axis in α‐syn‐related Central Nervous System (CNS) pathology. Environmental factors modify gut microbiota and intestinal permeability, influence gastrointestinal function and α‐syn aggregates. Motor disfunction in PD patients can be trigger from GI α‐syn aggregates which travel through vagus nerve to the substantia nigra *pars compacta* in the CNS. Alternative therapies, such as FMT or dietary fiber consumption, can ameliorate GI and motor dysfunction in PD patients. Similar effects have been reported in animal models of PD induced by rotenone. Image Created in BioRender. Velarde (2025) https://BioRender.com/t38o474. FMT, fecal microbiota transplant; GI, gastrointestinal; IP, intestinal permeability; SCFAs, short‐chain fatty acids; SN, substantia nigra; X, Vagus nerve.

Although aging is one of the main associated factors with GD and the progression of PD (Singh et al. 2023), lifestyle factors also play a crucial role in shaping the GM. In murine models, a low‐fiber diet and social isolation have been linked to increased intestinal permeability and proinflammatory responses, respectively, both of which contribute to the onset and progression of PD (Schmit et al. 2023; Singh et al. 2019). Furthermore, environmental factors such as chronic stress, exposure to pollutants, and microbial toxins have been shown to impact GD, gastrointestinal dysfunction, systemic inflammation, and metabolism balance. These disruptions can increase susceptibility to psychiatric and neurological disorders, including PD (Chaklai et al. 2024; Deng et al. 2024; Dodiya et al. 2020; Esteves et al. 2023). These findings lend support to a model of PD pathogenesis proposed by Baark, which suggests that an imbalance of the intestinal microbiome, including microbiota and metabolite production, induces a local inflammatory response and facilitates the formation of insoluble α‐syn aggregates in the gut. These aggregates are then hypothesized to spread in a prion‐like fashion along the vagal nerve to the brain (Figure 1) (Nakahara et al. 2023; Yang et al. 2024a). However, despite growing evidence supporting this model, the exact mechanism by which α‐syn aggregation is initiated in the gut remains unclear and is currently the focus of ongoing research.

The association between α‐syn and the GM is complex and has become a subject of growing scientific interest. Understanding this relationship could open new avenues to enhance microbial diversity and potentially modulate α‐syn function, thereby delaying PD progression. The primary aim of this narrative review is to present and analyze current evidence supporting the connection between GM and the α‐syn function in both preclinical and clinical PD research. This will help elucidate the mechanisms linking these factors and explore new therapeutic strategies to improve the quality of life of individuals with PD, as well as contribute to preventive approaches. This review focuses on describing microbial alterations that may impair α‐syn function in both experimental and human studies of PD. Additionally, it seeks to identify potential microbiota‐targeted interventions that could alleviate symptoms or delay disease onset.

## 2 Materials and Methods

### 2.1 Search Strategy

This narrative review included experimental designs, clinical studies, and intervention‐based studies. The selected information was organized according to specific research items. Following organization, data were analyzed and synthesized to draw conclusions and highlight the key findings. This analysis summarizes the current preclinical and clinical evidence about the relationship between the GM and the α‐syn function in PD, along with potential microbiota‐targeted interventions to alleviate the symptoms of PD.

This review was written according to the recommended methodology for the preparation of narrative reviews and applied the Scale for the Assessment of Narrative Review Articles (SANRA) to critically appraise the article produced (Baethge et al. 2019). The article selection process was conducted using Evidence for Policy and Practice Reviewer version 6 (EPPI v6). Although this software is primarily used for systematic reviews and meta‐analysis, it also enhances reliability and reproducibility in narrative review management. Article selection, analysis, and data organization were conducted by a single researcher, and the final manuscript was reviewed and approved by all co‐authors.

### 2.2 Record Discrimination, Inclusion, and Exclusion Criteria

**Figure 2:**
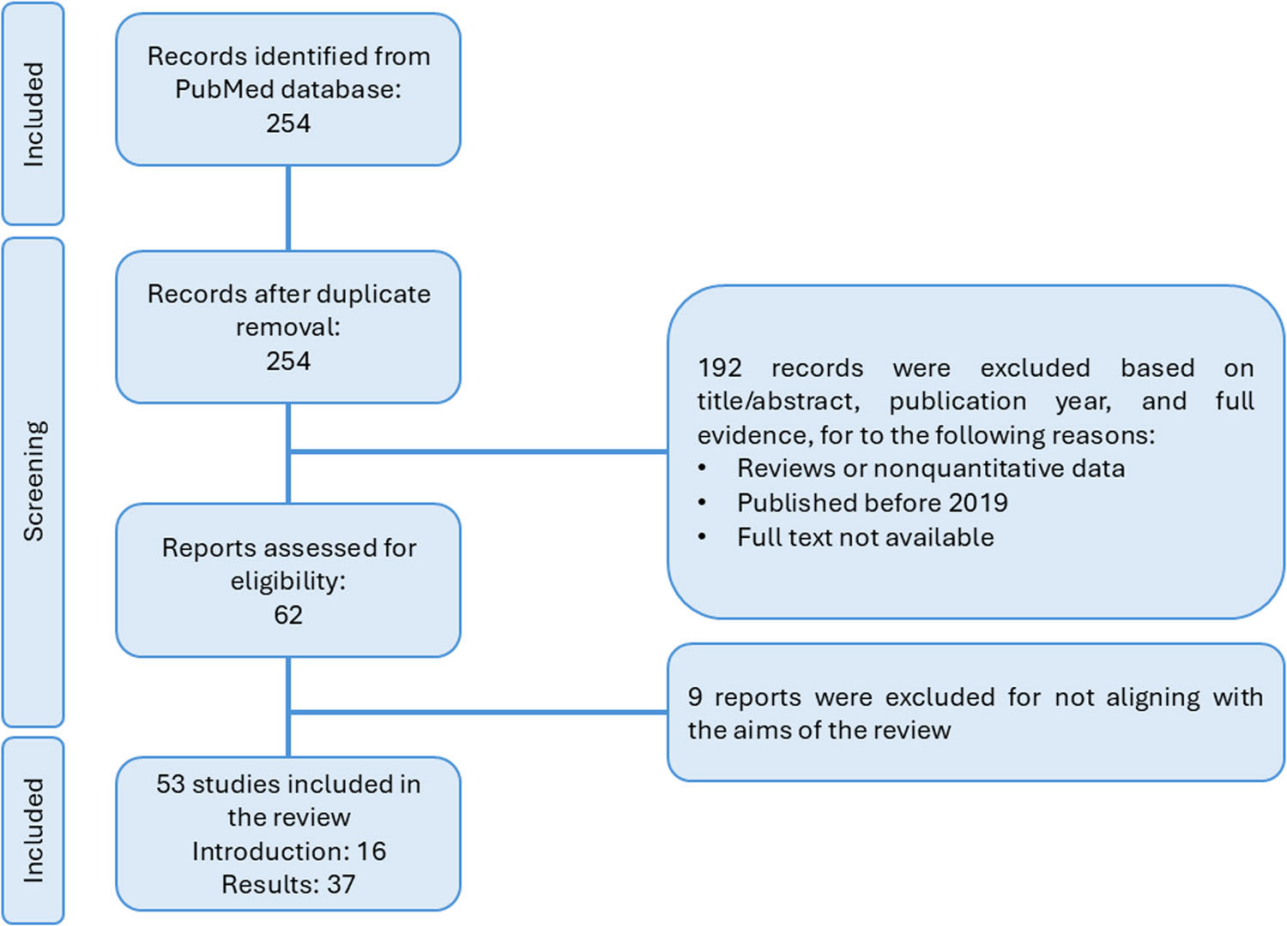
Methodological flowchart for this narrative review.

The article selection process is illustrated in Figure 2. A literature search was conducted in the EPPI Reviewer web using the PubMed database with search terms: (“gut microbiota”) AND (“alpha synuclein”) AND (“Parkinson”). Duplicate records were removed, resulting in 254 unique articles. The inclusion criteria were as follows: (1) Only research articles published between 2019 and 2024, (2) Articles only written in English, (3) Full‐text articles available (open access), and (4) Articles aligned with the specific aims of this review.

The exclusion criteria include: (1) review articles, protocols, or meta‐analysis; (2) Articles published before 2019 (unless deemed theoretically relevant); and (3) Articles without full‐text access.

## 3 Results

After applying these inclusion criteria, 192 articles were excluded. An additional eight articles were removed during the final screening because they did not directly address the review's main objectives. A total of 54 articles were included in the final synthesis, of which 17 supported the Introduction and 37 informed the Discussion section.

### 3.1 Data Extraction and Analysis From the Included Articles

The following data were extracted from preclinical studies: author, year, experimental model, assessment methodology, intervention, endpoints, outcome measures (Parkinsonism symptoms, description of GM, and relationships), and key findings. For clinical studies, the extracted data included the following: author, year, demographics (age and sex), sample size, intervention, follow‐up duration, assessment methodology (Parkinsonism symptoms, description of GM, and relationships), outcome measures, and key findings.

### 3.2 Information Organization

Each selected manuscript was critically analyzed, and the extracted information was synthesized and presented in alignment with the aims of this narrative review, aiming to provide a coherent and comprehensive understanding of how GM may influence α‐syn function in PD and the potential implications for treatment or prevention.

## 4 Discussion

### 4.1 The Gut Microbiota is Necessary for Motor and Gastrointestinal Dysfunction Associated With PD

Recent evidence supports the involvement of GM in PD pathophysiology. Here, we explore key studies demonstrating the essential role of GM in motor and gastrointestinal dysfunction. Findings from preclinical research in mice and *Drosophila melanogaster* have shown that gut microbiota is necessary to trigger progression (Liu et al. 2022a; Sampson et al. 2016). Antibiotic treatment has been reported to restore intestinal homeostasis and ameliorate disease progression, whereas untreated animals exhibit typical PD hallmarks, including a shortened life span, loss of dopaminergic neurons, progressive motor impairments, and increased reactive oxygen species activity (Sampson et al. 2016; Liu et al. 2022b).

Metagenomic analyzes have identified several taxa associated with motor and gastrointestinal dysfunctions observed in PD. At the phylum level, alterations in the GM have been linked to PD‐related dysfunctions. An increased abundance of Bacillota (formerly Firmicutes) has been positively correlated with motor dysfunction, while Actinobacteria levels show a negative correlation with colonic mucosa thickness. At the family level, pathogenic taxa such as Ruminococcaceae, Desulfovibrionaceae, and Faecalibacterium have demonstrated positive associations with motor impairments (Fang et al. 2024b). The presence of *Desulfovibrio*, *Sutterella*, and *E. coli* has been positively correlated with both motor and colonic dysfunctions (Liang et al. 2023; Yang et al. 2018). Conversely, certain beneficial genera exert protective effects. *Lactobacillus* has shown an inverse correlation with motor impairments, while both *Clostridium* and *Adlercreutzia* are negatively associated with colonic dysfunction (Yang et al. 2018).

In PD patients, positive correlations have also been found between motor symptom severity and the abundance of bacterial genera such as *Anaerotruncus*, *Clostridium*, and members of the Lachnospiraceae family (Heintz‐Buschart et al. 2018). In a transgenic model of overexpressing α‐syn, specific pathogen‐free (SPF‐ASO) mice exhibit severe motor dysfunctions compared to germ‐free α ‐syn overexpressing (GF‐ASO) mice (Sampson et al. 2016). In addition to motor symptoms, gastrointestinal alterations have also been observed. SPF‐ASO mice displayed decreased colonic motility, along with a reduced number and water content of stool pellets when compared to GF‐ASO counterparts (Sampson et al. 2016; Yang et al. 2018). Furthermore, studies employing rotenone‐induced models of PD have identified associations between a broad spectrum of gut pathogens and both motor and gastrointestinal impairments.

It is also important to highlight that intestinal motility depends, in part, on bacterial‐derived serotonin. Spore‐forming bacteria are crucial contributors to serotonin synthesis in the gut, and their abundance is significantly reduced in PD. This suggests that PD‐associated constipation may result not only from neuronal degeneration but also from depleted levels of tryptophan and serotonin, both of which are influenced by the composition of the GM (Wallen et al. 2022). These findings support the hypothesis of gene‐microbiome interactions influencing PD progression, through the production of bacterial metabolites and the modulation of host immune responses, which in turn can promote α‐syn expression or aggregation, leading to behavioral and functional impairments.

### 4.2 Proinflammatory and SCFAs‐Producing Bacteria in PD

The enteric nervous system (ENS), often referred to as the “second brain,” plays a vital role in regulating gastrointestinal motility, secretion, and barrier integrity. It is intricately connected with GM and the immune system through bidirectional communication pathways. Microbial metabolites can influence ENS activity by modulating neurotransmitter synthesis and enteric neuron function. In parallel, GD alters intestinal immune signaling, promoting local inflammation that impairs ENS integrity and contributes to neurodegeneration. The composition of gut microbiota in models and PD patients plays a crucial role in shaping immune responses and influencing neurodegenerative processes. In this section, we address the characteristic imbalance between increased proinflammatory bacteria abundance and the reduction of those capable of producing short‐chain fatty acids (SCFAs), key metabolites involved in gut and brain homeostasis.

The Enterobacteriaceae family, often enriched under dysbiotic conditions, has been reported to release endotoxins such as lipopolysaccharides (LPS), which activate the innate immune system and promote both intestinal and neuroinflammation (Fang et al. 2024b; Ortiz de Ora et al. 2024). This proinflammatory environment facilitates the aggregation and propagation of α‐syn from the gut to the brain (Sampson et al. 2016; Yang et al. 2018; Hegelmaier et al. 2020). At the class level, an increased abundance of Gammaproteobacteria has been positively associated with systemic inflammation and compromised intestinal barrier function, primarily through LPS production (Gorecki et al. 2019). Specific LPS‐producing species, such as *E. coli*, *Klebsiella*, and *Porphyromonas asaccharolytica*, have been identified in the GM of PD patients (Wallen et al. 2022). Moreover, *E. coli* has been shown to elicit a heightened pro‐inflammatory response in individuals with genetic susceptibility to PD (Liang et al. 2023).

Periodontal diseases have recently been associated with intestinal dysbiosis and neurodegenerative diseases due to their inflammatory mechanisms, including PD. In this regard, *Proteus mirabilis* can create a urease‐enriched environment that exacerbates inflammation by inducing cytokines such as TNF‐α and IL‐1β. This proinflammatory environment has been linked to alterations in α‐syn aggregate morphology (Grahl et al. 2023). Additional genera associated with increased proinflammatory responses in models of PD include *Rikenellaceae*, *Allobaculum* (Perez‐Pardo et al. 2018), *Subdoligranulum*, *Erysipelatoclostridium*, *Ruminococcus*, *Alloprevotella*, and *Flavonifractor* (Yang et al. 2024b). At the preclinical level, *Porphyromonas gingivalis* induced a pronounced proinflammatory response, upregulating IL‐17A, IL‐1β, and TNF‐α, which in turn led to microglial activation and dopaminergic neuron death in the SN*pc* (Feng et al. 2020). Although the mechanisms by which systemic inflammation promotes α‐syn aggregation remain unclear, GD has been strongly associated with the activity of mutant Leucine‐rich repeat kinase 2 (LRRK2), a kinase involved in immune signaling. Pathogenic variants like R1441G increase LRRK2 activity, leading to neuroinflammation and α‐syn aggregation. As LRRK2 is expressed in immune and intestinal epithelial cells, it may serve as a molecular link between dysbiosis, inflammation, and neurodegeneration in Parkinson's disease (Feng et al. 2020).

In contrast, SCFAs as butyrate, propionate, and acetate—produced by the fermentation of dietary fiber by commensal gut bacteria—exert neuroprotective and anti‐inflammatory effects (Hegelmaier et al. 2020; Vascellari et al. 2020). A reduction in SCFAs‐producing bacteria, including *Faecalibacterium*, *Bifidobacterium*, Lachnospiraceae, *Holdemanella*, *Howardella*, and *Anaerofustis*, was also observed in the GM of PD patients and animal models (Perez‐Pardo et al. 2018; Yang et al. 2024b; Vascellari et al. 2020). The depletion of these bacteria is associated with gut permeability, chronic inflammation, and enhanced α‐syn aggregation, all of which may contribute to PD pathogenesis and progression (Hegelmaier et al. 2020). Specifically, *Roseburia*, *Eubacterium*, *Ruminococcus*, and *Faecalibacterium prausnitzii* have been reported as significantly reduced in PD patients (Wallen et al. 2022). These bacterial species may represent a dysbiotic signature associated with PD. The reported mechanisms by which SCFAs promote neuroprotection include microglial maturation and a reduction in proinflammatory cytokines (Hegelmaier et al. 2020). However, most studies describe only associations between SCFA levels and motor or cognitive impairments (Vascellari et al. 2020), as the underlying mechanisms remain an emerging area of research.

### 4.3 Metabolic Dysregulation, Production of Bacterial Neurotoxic Metabolites, and Amyloid Proteins

In addition to compositional changes in GM, growing evidence highlights the metabolic consequences of dysbiosis in PD. GM generates a variety of metabolites that can cross physiological barriers and modulate host metabolism, immune responses, and neuronal integrity. Understanding how specific microbial products interact with host systems is key to understanding the GM's role in α‐syn aggregation and neurodegeneration in PD. This section explores recent findings related to metabolomic disruptions and microbiota‐derived neuroactive compounds implicated in disease progression.

Metabolic analyzes have demonstrated that intestinal dysbiosis disrupts the metabolism of key molecules involved in energetic metabolism―triglycerides, diglycerides―antioxidants like taurine, and structural components of the cell like phosphatidylcholines, in both the gastrointestinal tract and brain. In addition, several bacterial‐synthesized metabolites signaling of inflammatory and neurotoxic processes have been identified, including amine oxides, bile acids, and indole derivatives such as indoxyl sulfate, a byproduct of bacterial tryptophan metabolism. These metabolites have been identified as signaling molecules involved in inflammatory and neurotoxic processes. Notably, the branched‐chain amino acid valine and trimethylamine N‐oxide—both of bacterial origin—have been found to increase in the gut, plasma, and brain of PD animal models and patients, suggesting a systematic microbial signature associated with the disease (Morais et al. 2024).

Certain gut bacteria produce metabolites that can exert deleterious effects on neural function. For example, *Desulfovibrio*, a sulfate‐reducing bacterium, produces hydrogen sulfide (H_2_S). This neurotoxic metabolite can disrupt iron homeostasis, promote oxidative stress, and induce α‐syn aggregation, contributing to intestinal constipation and neurodegeneration associated with PD (Murros et al. 2021). Other bacterial species, such as *E. coli*, *Klebsiella*, and *P. gingivalis*, are known to produce amyloid proteins, such as curli fibrils, which can promote α‐syn aggregation. This enhances the prion‐like spread of α‐syn from the gut to the brain, exacerbating PD pathology (Liang et al. 2023; Wallen et al. 2022; Feng et al. 2020; Bhoite et al. 2022; Wang et al. 2021). Mechanistic studies have shown that *E. coli*‐related α‐syn aggregation is the result of anaerobic nitrate respiration, which leads to a cascade of oxidation reactions. These include dopamine oxidation, α‐syn misfolding, and its subsequent aggregation in intestinal epithelial cells (Ortiz de Ora et al. 2024).

Additional bacterial families, such as Rikenellaceae and Ruminococcaceae, have been associated with α‐syn accumulation in the colonic plexuses (Perez‐Pardo et al. 2018), a process that may serve as a potential biomarker for early PD diagnosis or risk assessment. However, the underlying molecular mechanism remains unclear. Other species associated with host proteostasis by protein aggregation include *Ralstonia sp*., *A. xylosoxidans*, *Pseudomonas sp*., and *Klebsiella pneumoniae*. This disruption in host proteostasis mechanisms caused by protein aggregation is related to cellular stress and neurodegeneration (Walker et al. 2024).

### 4.4 Interventions Targeting Gut Microbiota to Relieve or Prevent PD

Several therapeutic strategies aimed at modulating the GM—such as dietary interventions, fecal microbiota transplantation, and targeted bacterial therapies—are examined to alleviate or prevent PD symptoms. Understanding the mechanisms through which gut microbes influence neurodegeneration, including inflammation, immune modulation, and microbial metabolite production, offers promising avenues for the development of novel microbiome‐targeted therapies.

#### 4.4.1 Diet Modulation

GM is highly influenced by diet in humans and animal models. Table 1 summarizes findings from several interventional preclinical and clinical PD studies using diet—exogenous SCFAs, metabolite derived from roots such as ginger or turmeric, pre and probiotics, phenolic compounds present in coffee, wine, carrots, and other vegetal sources—to modulate the GM and other hallmarks of PD including motor and gastrointestinal symptoms, cell death, and α‐syn aggregation.

**Table 1:** Dietary compounds and their effects on PD models.

Intervention	Microbiota effect	Functional benefit	References
Fiber‐rich diet	↑ SCFA‐producing bacteria	↓ α‐syn ↑ Motor function ↑ Intestinal function	(Hegelmaier et al. (2020); Abdel‐Haq et al. (2022))
Sodium butyrate	Restores gut dysbiosis	↓ α‐syn in brain and colon ↑ Motor function ↑ Intestinal function	(Zhang et al. (2023a))
Pre/probiotics	Modulate gut microbiota	↓ α‐syn	(Goya et al. (2020))
Phenolic compounds	—	↓ α‐syn ↓ DA oxidation	(Balsamo et al. (2024); Yamasaki et al. (2020))
6‐Shogaol (ginger)	—	↑ Motor function ↓ Intestinal & brain α‐syn ↓ Inflammation	(Huh et al. (2023))
Ginseng	↑ *Lactobacillus*	↓ α‐syn ↑ TH expression	(Xu et al. (2024))
Curcumin	↑ Lactobacillaceae, Aerococcaceae, and Staphylococcaceae	↓ α‐syn ↑ Neuroprotection ↑ Motor function	(Cui et al. (2022))
β‐glucans (yeast‐derived)	↓ *Enterobacter*, *Desulfovobrio*, *Clostridium*, and *Akkermansia*	↑ Disintegrated α‐syn in plasma	(Raghavan et al. (2023))
α‐Ketoglutarate	↓ *Lachnospiraceae*	↑ α‐syn function ↓ DA degeneration	(Zhang et al. (2023b))
Trehalose	↑*Bacteroides*, *Lachnoclostridium*, and Defluviitaleaceae	↑ TH brain and intestine levels	(Pradeloux et al. (2024))
*Pediococcus pentosaceus*	↑*Muribaculaceae*, *Lachnospiraceae*, and Defluviitaleaceae. ↓Erysipelotrichaceae, Enterococcaceae, *Dubosiella*, and *Enterococcus*.	↑ Motor function ↓ α‐syn ↑ GABA & Nrf2	(Pan et al. (2022))
*FLZ (synthetic)	↓ *Akkermansia*	↑ Motor function ↑ Intestinal function ↓ Inflammation ↓ DA neuronal death	(Zhao et al. (2021))

*Note:* *, FLZ is a synthetic derivative of the natural compound schimosamide from marine sponges; DA, dopaminergic; TH, Tyrosine hydroxylase; Nrf2, nuclear factor E2‐related factor 2; ↓, decrease; ↑, increase.

#### 4.4.2 Fecal Microbiota Transplant (FMT)

FMT from healthy donors has shown promising results in treating PD‐associated dysbiosis. The general protocol for FMT consists of collecting, rehydrating, homogenizing, and filtering the fecal material. The resulting filtrate is then centrifuged, resuspended, and centrifuged again, yielding a final bacterial suspension with a defined concentration of colony‐forming units (CFUs). This suspension is administered orally using formulated pellets (Fang et al. 2024b).

FMT can restore the diversity and functions of GM and improve motor symptoms. On the other hand, FMT reduces intestinal barrier disruption, endotoxemia, and systemic inflammation (Sampson et al. 2016; Fang et al. 2024b). FMT may also protect against PD by suppressing α‐syn expression, increasing the number of survival dopaminergic neurons, and inactivating proinflammatory signaling pathways, particularly those involving neuroglial activation (Zhong et al. 2021). Restoring the balance of the intestinal microbiota composition through FMT could alleviate, and potentially reverse, the intestinal pathological changes and PD‐like phenotypes induced by opportunistic microorganisms such as *E. coli* in mouse models (Liang et al. 2023). Despite its potential, further studies are needed to optimize FMT protocols and assess their long‐term efficacy. Additionally, FMT includes not only beneficial microorganisms but also opportunistic species―including bacteria, viruses, archaea, and other microbes― even when the sample is obtained from healthy donors. This complexity may complicate the interpretation of results.

#### 4.4.3 Therapies for Targeting Specific Bacteria

Targeted elimination of harmful bacteria using antibiotics or bacteriophages may offer therapeutic benefits. For instance, removing *Desulfovibrio* or *Proteus mirabilis* may reduce the production of neurotoxic metabolites and inflammation (Grahl et al. 2023; Murros et al. 2021). *P. mirabilis* has been linked to dopaminergic neuron damage, inflammation, and aggregation of α‐syn in both the brain and the colon (Choi et al. 2018). Targeting Rikenellaceae and Ruminococcaceae families may benefit intestinal barrier integrity (Perez‐Pardo et al. 2018).

Moreover, the increase of *Clostridium*, *Bifidobacterium*, and *Prevotella corporis* has been suggested as a potential therapy to enhance colonic function, physical function, and suppress protein aggregation (Yang et al. 2018; Perez‐Pardo et al. 2018; Walker et al. 2024). Conversely, targeting *Akkermansia muciniphila*, which promotes α‐syn aggregation and oxidative stress, could offer therapeutic value (Amorim Neto et al. 2022). However, any such strategies must consider the potential impact on overall microbiota diversity.

### 4.5 Integrated Discussion and Evidence Synthesis

In this review, we analyze the key role of GM in the pathogenesis of PD in preclinical and clinal research, specifically in how microbiota may influence the α‐syn function. PD is a multisystemic, progressive, and neurodegenerative disorder with a wide range of symptoms, including intestinal dysfunction, microbiota dysbiosis, systemic inflammation, α‐syn misfolding and aggregation in the gut and brain, and neuronal death. As we saw, the increasing body of evidence demonstrates an imbalance of GM in models and PD patients, which is strongly associated with motor and non‐motor symptoms. This fact is supported by preclinical research where chronic treatment with antibiotics depletes motor and non‐motor dysfunction in rotenone‐treated mice (Sampson et al. 2016). However, these observations should be carefully considered in human interventions due to the heterogeneity of protocols, including dosage, type of administration, and animal model.

The vagus nerve has been identified as the main gate by which α‐syn spreads out to the brain, contributing to PD progression. However, this is not consistent with the report by Shan et al (Shan et al. 2022)., since mice subjected to subdiaphragmatic vagotomy before the administration of 1‐methyl‐4‐phenyl‐1,2,3,6‐tetrahydropyridine (MPTP) did not reduce the aggregation of α‐syn in the colon and did not reverse the reduction of tyrosine hydroxylase and the dopaminergic transporter in the striatum. This suggests that the subdiaphragmatic vagus nerve does not play a role in the MPTP‐induced neurotoxicity in the brain and colon. A standardized protocol is needed for surgical interventions to reduce the discrepancy regarding the role of the vagus nerve in the prevention/progression of PD.

Studies often use 16S rRNA gene sequencing to assess GM composition. However, this method provides only relative data, showing bacterial proportions within a community rather than absolute abundances. For example, if a study reports a Bacillota/Bacteroidetes ratio of 60% and 40%, respectively, this reflects their relative proportions but does not indicate their absolute quantities. If the absolute abundance of Bacillota decreases but that of Bacteroidetes decreases, the relative ratio may remain unchanged, potentially masking important shifts in total microbial biomass or specific ecosystem configurations associated with disease. To address this limitation, 16S rRNA sequencing can be complemented with qPCR targeting specific bacterial groups, as demonstrated by Murros et al (Murros et al. 2021)., who found that a higher absolute abundance of *Desulfovibrio* was associated with greater PD severity.

Additionally, single‐cell RNA sequencing (sc‐RNA‐seq) offers a broader framework to characterize bacterial phenotypic heterogeneity in PD. Within isogenic bacterial populations, single cells can exhibit variation in transcriptional states due to gene regulation, chromosomal replication, and microenvironmental factors—including interspecies competition, physicochemical gradients, host interaction, or stress responses—enabling functional and phenotypic specialization. In this sense, sc‐RNA‐seq provides the ability to determine which species express specific genes and how these functions are distributed among individual bacterial cells (Pountain and Yanai 2025). In the context of PD, this emerging concept suggests that microbial involvement in disease pathogenesis cannot be understood solely through community composition or bulk abundance. Instead, subpopulations of bacteria may differentially produce neuroactive or inflammatory metabolites, interact with the intestinal epithelium, or influence α‐syn misfolding through localized molecular signaling. Thus, integrating single‐cell approaches could redefine how we conceptualize microbial dysbiosis in PD.

Although α‐syn pathology has been repeatedly linked to gut‐derived factors, the exact trigger of its misfolding in the gastrointestinal tract remains unclear. Most of the mechanistic evidence comes from transgenic mouse models, which may not fully recapitulate the complexity of human PD. Furthermore, inconsistencies in the taxa reported across different human studies—influenced by geographic, dietary, and methodological variability—highlight the need for harmonized study protocols. Moreover, it is essential to consider alternative perspectives regarding bacterial‐amyloid protein interactions. For instance, Walker et al (Walker et al. 2024). demonstrate that the folding of neurodegeneration‐associated amyloid proteins does not occur through a direct mechanism. Instead, it happens indirectly by disrupting the host's proteostasis.

On the other hand, therapeutic strategies that aim to restore microbial balance or reduce microbial‐derived toxicity show great promise. The heterogeneity of PD symptomatology and individual microbiota profiles suggest that personalized approaches may be necessary to achieve consistent therapeutic outcomes. Interventions such as fiber‐rich diets, sodium butyrate supplementation, probiotics, phenolic compounds, and botanicals like ginger, curcumin, and ginseng have demonstrated protective effects in animal models by modulating gut bacteria, reducing inflammation, and preventing dopaminergic neuron loss (Goya et al. 2020; Huh et al. 2023; Cui et al. 2022). At present, most of these interventions have only been assessed in rodent models, with limited translation to clinical trials. Moreover, the dose, timing, and combinatory effects of these interventions remain insufficiently explored.

## 5 Conclusions

In conclusion, GM plays a crucial role in modulating α‐syn function in PD. GM dysbiosis may contribute to α‐syn aggregation and propagation, neuroinflammation, and neurodegeneration. Modulating GM through dietary interventions, FMT, or therapies targeting specific bacteria could be a promising strategy to alleviate or prevent PD. Further research is needed to fully understand the complex interactions between the GM and α‐syn function and to develop effective and safe microbiota‐targeted therapies for PD.

## 6 Future Directions

Future research should prioritize the identification of microbial signatures predictive of PD onset and progression, with a particular focus on longitudinal human studies. Additionally, efforts are needed to clarify the causal relationships between specific taxa and neuropathological outcomes, using gnotobiotic and mechanistic models. The development of standardized protocols for microbiome‐based interventions, including FMT and personalized probiotics, will be critical. Furthermore, multi‐omics approaches that integrate metabolomics, transcriptomics, and proteomics with microbiome data could yield powerful insights into the host‐microbiota interaction. The translation of these discoveries into clinical practice will depend on large‐scale trials that evaluate the efficacy, safety, and long‐term outcomes of microbiota‐modulating therapies in PD populations.

## Author Contributions

**I. Daniel Salinas‐Velarde:** conceptualization (lead), methodology (lead), investigation (lead), writing – original draft (lead), writing – review and editing (equal). **Juan Manuel Donaciano‐Domínguez:** investigation (supporting), writing – original draft (supporting), writing – review and editing (equal). **Rigoberto Oros‐Pantoja:** resources (supporting), writing – review and editing (equal), supervision (equal). **José Félix Aguirre‐Garrido:** validation (supporting), writing – review and editing (equal), supervision (equal). **Rina María González‐Cervantes:** writing – review and editing (supporting). **Jacobo Esteban Munguía‐Cervantes:** writing – review and editing (supporting). **Modesto Gómez‐López:** writing – review and editing (supporting). **Jaime Bustos‐Martínez:** writing – review and editing (equal), supervision (equal), writing – review and editing (equal), supervision (lead). **Alexandra Estela Soto‐Piña:** conceptualization (supporting), methodology (supporting), validation (lead), writing – original draft (supporting), writing – review and editing (equal), supervision (lead).

## Ethics Statement

The authors have nothing to report.

## Conflicts of Interest

The authors declare no conflicts of interest.
